# Rapid homeostatic plasticity and neuropsychiatric therapeutics

**DOI:** 10.1038/s41386-022-01411-4

**Published:** 2022-08-22

**Authors:** Ege T. Kavalali, Lisa M. Monteggia

**Affiliations:** Department of Pharmacology and the Vanderbilt Brain Institute, Vanderbilt University, Nashville, TN 37240-7933 USA

**Keywords:** Neuroscience, Synaptic plasticity

## Abstract

Neuronal and synaptic plasticity are widely used terms in the field of psychiatry. However, cellular neurophysiologists have identified two broad classes of plasticity. Hebbian forms of plasticity alter synaptic strength in a synapse specific manner in the same direction of the initial conditioning stimulation. In contrast, homeostatic plasticities act globally over longer time frames in a negative feedback manner to counter network level changes in activity or synaptic strength. Recent evidence suggests that homeostatic plasticity mechanisms can be rapidly engaged, particularly by fast-acting antidepressants such as ketamine to trigger behavioral effects. There is increasing evidence that several neuropsychoactive compounds either directly elicit changes in synaptic activity or indirectly tap into downstream signaling pathways to trigger homeostatic plasticity and subsequent behavioral effects. In this review, we discuss this recent work in the context of a wider paradigm where homeostatic synaptic plasticity mechanisms may provide novel targets for neuropsychiatric treatment advance.

## Introduction

Synaptic plasticity is generally considered within the context of synaptic adaptations where, synaptic strength and subsequent neuronal activity are modified in the direction of prior “conditioning” stimulation. For instance, in classical long-term potentiation (LTP) the strong stimulation or pairing—coincidence of presynaptic activation with postsynaptic depolarization—results in strengthening of a particular synapse. Along the same lines, a weaker stimulation sustained over a longer period causes a decrease in synaptic strength of a specific synapse resulting in long-term depression (LTD). A key feature is that in both cases the changes in synaptic strength far outlast the duration of this initial induction phase, possibly for days and weeks. Since the early 1970s, the mechanisms underlying these forms of Hebbian plasticities (initially LTP and later LTD) have received intense attention from neurophysiologists partly because their properties satisfied the earlier postulate by Donald Hebb as synaptic correlates for learning and memory [1].

However, decades prior to the discovery of LTP, another form of plasticity had been widely observed and studied albeit predominantly in the peripheral nervous system. It was well-documented that target membranes show hyperexcitability following the denervation or other disruption of their nervous input [2]. In the neuromuscular junction, this form of plasticity could be triggered after denervation of incoming motor axons and manifested as an increase in sensitivity of muscle tissue to acetylcholine, mediated by an upregulation of acetylcholine receptors [3]. Studies in the last two decades have shown that a similar upregulation of postsynaptic receptors occurs at mammalian central synapses following suppression of neuronal activity, uncovering a robust form of synaptic plasticity that aims to maintain activity levels in neuronal circuits [4, 5]. Specifically, chronic (1–2 day) blockade of action potential firing augments trafficking of GluA1 and GluA2 AMPA receptor subunits to postsynaptic sites, thus increasing the sensitivity and thus magnitude of responses to released glutamate [6].

In many aspects these “homeostatic plasticities” resemble LTP or LTD as they largely rely on similar mechanisms targeting presynaptic fusion machinery and/or postsynaptic receptor trafficking machinery to up- or downregulate synaptic strength, although possibly with some key differences in the specific molecular players [7–9]. However, homeostatic plasticities differ in one critical aspect, they work against the tide to counter the synaptic changes imposed by the environment to preserve synaptic strength and the overall dynamic range of activity, and therefore do not follow but oppose the direction of environmental inputs. For example, synapses are strengthened in response to suppression of activity or neurotransmission, alternatively synapses are weakened in response to increases in activity or augmentation of neurotransmission. A key feature is that these forms of plasticities act globally, not necessarily in a synapse specific manner, although some synapse specific, more local, forms of such plasticity have been observed [8]. Homeostatic synaptic plasticities act independently of Hebbian plasticities, as they aim to preserve the dynamic range—in terms of action potential firing rates and synaptic weights—and thus act in a permissive manner to enable LTP or LTD to encode information [9].

## Types of homeostatic plasticity

### Homeostatic postsynaptic synaptic scaling

In pioneering work, Turrigiano and colleagues observed that sustained suppression of activity with the voltage gated Na^+^ channel blocker TTX, or enhancement of activity via blockade of GABA receptors (e.g., with bicuculline) for a period of 24 to 48 h, resulted in up- or down-regulated synaptic strength respectively by changing the amplitudes of unitary synaptic events. This change was mediated by alterations in trafficking of postsynaptic AMPA receptors and occurred in a “mathematically” specific fashion where each synapse was multiplied by a specific “scaling factor”. Quantitatively, these plasticities preserve relative strengths of synapses and thus result in minimal alterations in the information stored as “synaptic strength”, and rather enable a re-arrangement of synaptic weights to support further changes in synapse specific strength.

**Fig. 1:**
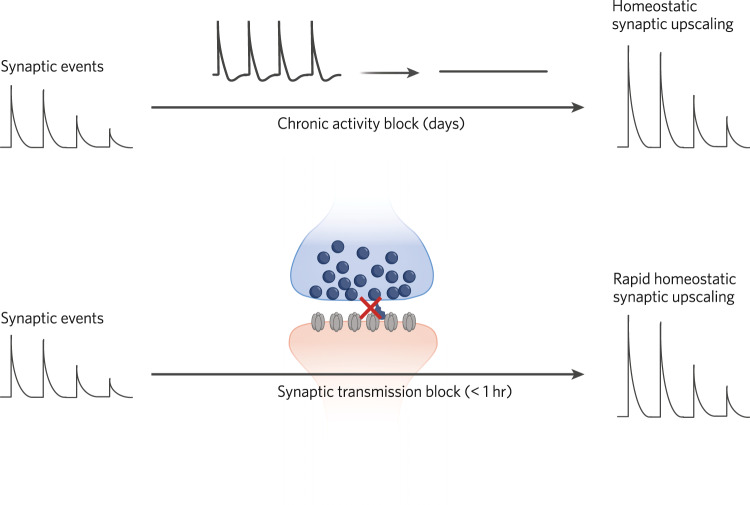
Rapid versus slow homeostatic plasticity. Figure depicts the impact of synaptic transmission blockade on the rapidity of homeostatic plasticity. While classical experiments relying on activity blockade (via blockade of voltage-gated Na^+^ channels or postsynaptic AMPA receptors) demonstrated a slow process that occurs over days, block of synaptic NMDA receptor facilitates plasticity down to hours or less.

More recent studies have demonstrated that direct manipulation of synaptic transmission—independent of neuronal activity via manipulation of postsynaptic receptors or presynaptic release machinery—can elicit homeostatic plasticity albeit in a faster time scale (within a few hours rather than days) as seen in response to activity blockade [8, 10, 11] (Fig. 1). These studies highlighted the potential role of spontaneous neurotransmission in maintenance of baseline synaptic strength, as blockade of spontaneous neurotransmission appeared to be essential to facilitate the pace of homeostatic synaptic scaling.

Spontaneous neurotransmitter release is a common feature of synapses throughout the nervous system, but its function is poorly understood. Since its initial discovery by Fatt and Katz [12, 13], it has been unclear why synapses—dedicated to information transfer and processing with high temporal and spatial precision—give rise to spontaneous vesicle fusion events and neurotransmitter release. A few studies have examined spontaneous neurotransmission and demonstrated its potential purely electrical function to regulate action potential firing typically in neurons with high membrane resistance [14]. However, recent studies have demonstrated that spontaneous neurotransmitter release can trigger biochemical signaling to suppress dendritic protein translation machinery locally. This suppression helps maintain baseline synaptic strength by stabilizing receptor composition at synapses and acts as a specific regulator of postsynaptic sensitivity to neurotransmitters [8, 11]. Therefore, once inhibited, the decrease in spontaneous neurotransmission leads to an upregulation of dendritic protein translation and an increase in levels and secretion of the neurotrophic factor brain-derived neurotrophic factor (BDNF), as well as postsynaptic receptor numbers, leading to potentiation of neurotransmission [11, 15]. However, unlike the blockade of activity (or action potentials), inhibition of glutamatergic NMDA receptors can increase the amplitude of excitatory postsynaptic currents within mere hours. Importantly, while the impact of activity blockade on synaptic efficacy relies on transcriptional regulation and occurs over a day, homeostatic synaptic plasticity after blockade of NMDA receptors solely depends on protein translation and occurs rapidly. Overall, these findings point to two surprising facts. First, they predict that NMDA receptors are active near resting membrane potentials in response to spontaneous glutamate release, despite their reduced ionic conductance due to Mg^2+^ block. This initial prediction was later directly validated in hippocampal neurons in vitro as well as in brain slices using electrophysiological and optical imaging approaches [16, 17]. Second, the observation that amplitudes of synaptic responses increase rapidly, within an hour after NMDA receptor blockade, stands in striking contrast to earlier reports that chronic blockade of neuronal firing by TTX leads to slow up-scaling of mEPSC amplitudes [4]. This observation is critical as it directly pertains to the rapid action of the NMDA receptor blocker ketamine as an antidepressant [10, 11].

### Homeostatic presynaptic plasticity

In contrast to postsynaptic scaling, homeostatic or negative feedback responses to chronic alterations in network activity may also elicit presynaptic forms of plasticity [18, 19]. These presynaptic modifications typically occur via retrograde signaling targeting presynaptic neurotransmitter release machinery following postsynaptic signal transduction events [19, 20]. However, presynaptic alterations in synaptic strength—largely based on changes in presynaptic release probability—do not follow the mathematical “scaling” rule as they do not necessarily preserve the relative strengths of postsynaptic efficacy among a set of synapses but rather alter the dynamics of neurotransmission during bursts of activity. Due to this distinction between pre- versus postsynaptic forms of homeostatic plasticity, alterations in presynaptic release probability do not only up- or downregulate synaptic strength but also alter information processing dynamics of a synapse [21, 22].

In mammalian synapses acute block of postsynaptic AMPA receptors or genetic deletion of GluA4 AMPA receptors [19, 20, 23] may trigger a presynaptic form of homeostatic plasticity. Presynaptic forms of homeostatic plasticity seen in mammalian systems may have substantial commonalities with similar mechanisms extensively studied in the Drosophila Neuromuscular Junction [24]. In addition to pharmacological block of postsynaptic excitatory neurotransmitter receptors, direct manipulations of synaptic vesicle fusion machinery components, in particular at the neuromuscular junction, leads to suppression of neurotransmitter release and elicits similar homeostatic plasticity responses [25, 26]. Furthermore, genetic deletion of presynaptic voltage gated P/Q type Ca^2+^ channels may also elicit changes in presynaptic output driven by transcriptional regulation [27]. There is also increasing evidence that although global manipulations altering neuronal activity provided the initial insight into homeostatic plasticity, even subtle maneuvers that directly interfere with presynaptic function can lead to robust homeostatic synaptic plasticity [11, 28].

## Linking cellular excitability and synaptic scaling

Homeostatic plasticity mechanisms were initially thought to operate via targeting cellular excitability patterns, in particular the rate of neuronal action potential firing [4, 29, 30]. It has been postulated that the firing rate in a single neuron may act as the set point to adjust the strength of synaptic input onto that neuron. However, recent studies have provided a more complex picture of the mechanisms underlying this firing rate adaptation, where regulation of neuronal firing rates may or may not go hand in hand with plasticity of synaptic input onto a particular neuron. For instance, neuronal activity patterns can be altered in a homeostatic manner directly by regulating ion channel molecular composition [31].

While direct regulation of ion channel properties may provide a means for plasticity independent of synaptic strength, studies have also demonstrated that synaptic strength can be regulated directly independent of potential changes in neuronal action potential firing. In particular alterations in spontaneous neurotransmitter release, which is driven independent of presynaptic action potentials, have emerged as a key determinant. Several studies have demonstrated that spontaneous release operates via dedicated mechanisms for its maintenance and regulation. In addition, it is segregated from action potential evoked neurotransmission in regards to its postsynaptic targets [32–38]. Studies in multiple systems—including hippocampal neurons, embryonic spinal cord, and Drosophila neuromuscular junction—have revealed a critical role for sensing of baseline neurotransmission, primarily driven by spontaneous release independent of activity, in this process [39]. Thus, the regulation of spontaneous release plays a specific role in synaptic scaling based on recent experiments performed in the spinal cord and Drosophila neuromuscular junction as well as mammalian central synapse systems. For instance, recent work showed that directly targeting spontaneous release-specific machinery without altering network activity or evoked neurotransmission was sufficient to trigger plasticity [28, 40]. In addition, using optogenetic stimulation to clamp cell wide activity levels while manipulating neurotransmitter input could also trigger plasticity [41, 42]. Overall, these studies suggest an autonomous role for spontaneous release or sensing neurotransmitter input, rather than activity per se, in the regulation of synaptic strength.

## Sensors that activate homeostatic plasticity

Neuronal and synaptic signaling have been often associated with activity. Increases in activity levels typically give rise to elevations in local or global calcium signals which can exert effects on signal transduction pathways. While Hebbian plasticities typically require synapse specific “local” calcium signals, global elevations in calcium during strong increases in activity may trigger biochemical events to suppress activity or synaptic strength [43]. In this context, detecting neuronal or synaptic “silence” poses a puzzle. While it is relatively straightforward to envision how neurons sense activity, it is more difficult to formulate models that account for sensing neuronal silence. Recent research in homeostatic plasticity mechanisms has addressed this conundrum by demonstrating that neurons continue signaling under the suppression of activity. Importantly, this form of signaling has key qualitative differences and cannot be simply accounted by a down regulation or a miniature version of activity dependent signaling [44]. For example, studies have proposed several potential sensors that mediate this form of “resting” signaling, which include mitochondrial function [45], calcium signals mediated by voltage gated T-type or L-type calcium channels that are active near resting membrane potentials [46–50]. These “resting” calcium signals in turn desupress protein translational and/or gene transcription mechanisms. Here, as mentioned previously, resting NMDA receptor activity driven by spontaneous release has received particular attention as it is primarily targeted by ketamine and other NMDA receptor blockers to trigger rapid antidepressant action [10, 11, 51]. However, reduction in baseline calcium levels can also elicit signaling. For instance, prolonged blockade of synaptic activity decreases resting calcium levels in neurons and inhibits the calcium-calmodulin dependent phosphatase Calcineurin. Suppression of Calcineurin activity promotes retinoic acid (RA) synthesis leading to an increase in excitatory synaptic transmission coupled with a decrease in inhibition [52].

## Impact of neuropsychiatric therapeutics on homeostatic plasticity

In a recent study, we investigated a causal link between multiplicative synaptic up-scaling and fast-acting antidepressant effects and proposed that this form of synaptic plasticity is a major synaptic substrate required for the efficacy of rapid antidepressant action. As indicated above, multiplicative synaptic up-scaling can be elicited over days in response to activity block, but rapidly within hours after block of synaptic inputs, in particular NMDA receptor mediated transmission, and results in an increase in synaptic strength via postsynaptic insertion of AMPARs in quantities that are proportional to the amount of pre-existing AMPAR clusters in a synapse. In this way, up-scaling preserves relative strengths of synapses in order not to disrupt information storage while enabling global control of synaptic activity [4, 22, 53].

In earlier work, we demonstrated that protein translation, in particular synthesis of new AMPARs, is a critical mechanism to induce synaptic scaling downstream of acute resting NMDA receptor activity block or transient eukaryotic elongation factor 2 kinase (eEF2 kinase or eEF2K) inhibition [8, 10, 11, 17, 54]. Thus, transient inhibition of tonic eEF2 kinase (a Ca^2+^-calmodulin-dependent kinase with a high affinity for Ca^2+^-calmodulin that renders it active at low levels of Ca^2+^) activity leads to the recruitment of AMPARs within the postsynaptic specializations that results in the rapid (within an hour) induction of synaptic up-scaling [11]. In this way, acute eEF2 kinase inhibition, which can be elicited by ketamine mediated NMDAR block, decreases eEF2 phosphorylation, augments local protein synthesis and produces synaptic potentiation that drives the rapid antidepressant effects [10]. Here, it is important to note that in contrast to block of NMDA receptors, AMPA receptor block does not elicit these effects, while their activity is actually required for the plasticity and behavioral effects [10, 11]. Previous studies have also reported a role for RARα as a regulator for AMPAR translation and homeostatic plasticity in hippocampal neurons [50, 55, 56]. In recent work, we confirmed that acute activation of RARα triggers synaptic scaling in hippocampal neurons and in turn demonstrated that this activity can mediate antidepressant-like effects in mice. Although RA signaling acting through RARα mediates a similar form of synaptic plasticity in the hippocampus as acute eEF2 kinase inhibition, these pathways are molecularly independent. RARα contains a mRNA-binding domain and represses protein translation of target mRNAs including GluA1. When RARα is activated through the binding of RA or an RARα agonist, it reduces mRNA binding and de-represses translation [55–57]. In contrast, eEF2 kinase and eEF2 regulate peptide elongation during protein translation, which constitutes a distinct mechanism for protein expression de-suppression compared to the RARα-pathway [58].

**Fig. 2:**
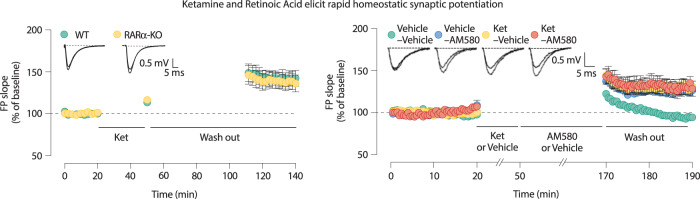
Rapid homeostatic plasticity elicited by ketamine and retinoic acid. Preclinical studies have demonstrated that both ketamine and retinoic acid can elicit a rapid form of homeostatic plasticity. Ketamine and retinoic acid have been also shown to trigger a rapid antidepressant-like effect in animal models. Left: Field excitatory postsynaptic potentials (fEPSPs) in 20 μM ketamine-treated wild type and RARα-knock-out hippocampal slices. Initial field potential (FP) slopes were plotted as a function of time. Inset, representative wave forms from wild type and RARα-knock-out slices before and after 30 min. rapid treatment. Right: fEPSPs were recorded from wild type hippocampal slice treated with AM580, a RARα agonist, in the presence or absence of ketamine pretreatment. After 20 min of stable baseline measurement, 20 μM ketamine or vehicle were applied for 30 min. After these pretreatments, 20 μM AM580 was applied for 2 h and then fEPSP responses were recorded for 20 min. Note that effects of ketamine and AM580 treatments occlude each other suggesting a common endpoint. Inset, representative wave forms before and after treatment (Modified from [58]).

Previous evidence has shown that treatment with an AMPAR antagonist blocks the antidepressant action of ketamine in preclinical models [59]. The finding that ketamine, by blocking NMDARs, triggers protein synthesis dependent insertion of new AMPARs and subsequent synaptic potentiation in Schaffer collateral to CA1 pyramidal neuron synapses provided a mechanistic link and explanation for the findings. Further studies showed that AMPAR antagonists inhibit the ketamine-mediated synaptic potentiation and strengthened the link between the requirement for synaptic potentiation and antidepressant action [10, 11]. We also found that RA signaling follows a similar sequence of events and requires AMPARs for the synaptic potentiation and antidepressant-like behavioral effects. However, the contribution of specific AMPAR subunits is different between these two drugs. Our findings showed RARα activation increases GluA2-lacking GluA1 homomers in the CA1 region of the hippocampus—in agreement with earlier work [55]—whereas ketamine increases GluA2-containing AMPARs [11]. Given that AMPAR subunit compositions alter Ca^2+^ permeability and channel conductance [60], the cellular response of RARα activation and ketamine may be different. Nevertheless, these findings support the theory that two parallel forms of signaling can converge on a synaptic end point by eliciting a homeostatic form of plasticity that is required for rapid antidepressant action (Fig. 2). Importantly, this work also demonstrated that ketamine administration indeed elicits multiplicative synaptic scaling in vivo using immunohistochemical analysis to visualize surface expression of GluAs via antibodies against their extracellular epitopes.

In these preclinical studies, complementary pharmacological and genetic approaches probed the intersection of ketamine- and RA-mediated signaling pathways and showed that multiplicative synaptic scaling is elicited independently by the two pathways but nevertheless triggered the same antidepressant-like behaviors. The convergence of two distinct biochemical pathways on synaptic scaling and antidepressant-like behaviors points to the potential role of synaptic scaling in the hippocampus to alter neuronal circuits and modulate the function of other brain regions. As global augmentation of synaptic efficacy via synaptic upscaling in the hippocampus appears to be causally linked to the behavioral effects of ketamine, this form of homeostatic synaptic plasticity represents a previously unexplored target for the treatment of major depressive disorder.

An alternative model proposed to account for ketamine action posits that ketamine’s primary targets are the NMDA receptors on inhibitory interneurons within the prefrontal cortex or hippocampus. According to this model, ketamine blocks these NMDA receptors and suppresses inhibition, thus leading to disinhibition and increase in the activity of excitatory pyramidal neurons [61, 62]. This model does not incorporate homeostatic synaptic plasticity as a mechanism, and it is hard to reconcile with the persistent clinical observation that a closely related NMDA receptor blocker memantine does not elicit rapid antidepressant effects in the clinic despite its ability to trigger disinhibition similar to ketamine [63, 64].

## Transition of acute homeostatic plasticity to long term effects

**Fig. 3:**
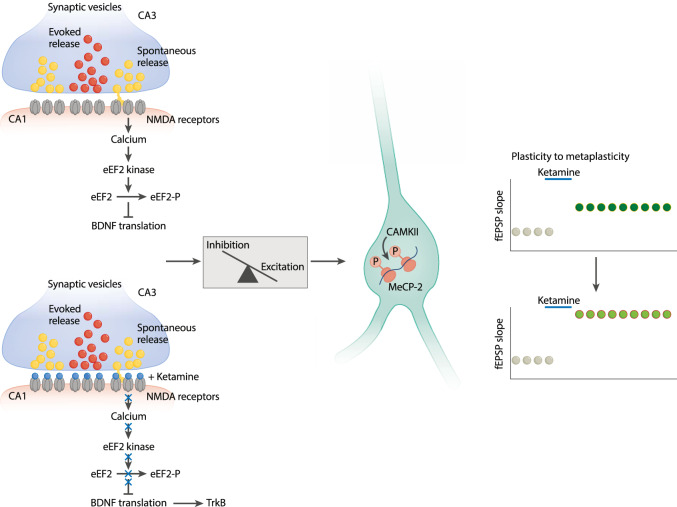
How may rapid homeostatic plasticity transition to chronic effects? Figure depicts the transition of rapid homeostatic plastic synaptic potentiation towards more sustained effects via transcriptional processes. In this particular example, rapid plasticity elicited by ketamine triggers phosphorylation of the transcriptional regulator MeCP2, which in turn triggers metaplasticity of the ketamine effect (i.e., second ketamine administration gives rise to an augmented response).

As indicated above, ketamine triggers a rapid form of homeostatic plasticity and manipulation of this plasticity disrupts the associated antidepressant-like effects. Importantly, the time course of these rapid homeostatic plasticities matches the response time course to ketamine administration seen in patients [10]. However, it has been unclear how ketamine action gives rise to long-lasting changes that persist for days or weeks. In the literature there is limited insight into mechanisms that maintain synaptic parameters altered by initial homeostatic plasticity events beyond the potential role of transcriptional regulation [46]. For instance, it is unclear how initial local translation regulation in dendrites transitions to transcriptional mechanisms that are likely needed for the sustained effects. It is also unclear whether the induction of homeostatic plasticity that leads to downstream effects is required to maintain the behavioral responses. To address this issue, we recently examined the role of key transcription-dependent mechanisms that respond to alterations in neuronal activity patterns—Methyl-CpG-binding protein 2 (MeCP2), cAMP response element-binding protein, and myocyte enhancer factor 2C—for their potential roles in persistent synaptic and behavioral changes [65]. Assessing the role of these factors in long-term antidepressant effects of ketamine revealed a specific role for MeCP2. MeCP2 is a transcriptional regulator originally identified as a protein that binds to methylated CpG sites and interacts with other transcriptional complexes. Existing literature shows MeCP2 is regulated by phosphorylation at Serine 421(S421) in response to BDNF upregulation as well as neuronal activation. We found acute subanesthetic ketamine increased MeCP2 Ser421 phosphorylation in the hippocampus 7-days after treatment but not in the initial hours after injection when the rapid antidepressant response occurs. Moreover, ketamine’s sustained antidepressant action was not observed in mutant *Mecp2* S421A knock-in mice although the rapid antidepressant effects were observed. These experiments demonstrate the sustained antidepressant effects of ketamine are dependent on MeCP2 S421 phosphorylation (Fig. 3).

How is MeCP2 Ser421 phosphorylation required for the long-term antidepressant effects of ketamine but not the acute effects? We propose a working model in which the initial translation dependent effects of ketamine [10, 11] are maintained via activation of a positive feedback loop. This model extends our previous work on the cellular mechanism of ketamine action in which we proposed that ketamine blocks postsynaptic NMDA receptors that are activated by spontaneous glutamate release, thereby decreasing the calcium influx. This results in inactivation of the calcium-calmodulin dependent kinase, eEF2 kinase, and ultimately dephosphorylation of its sole target eukaryotic elongation factor 2 (eEF2) which de-suppresses protein translation, including translation of BDNF. This increase in BDNF binds to and activates TrkB and results in the subsequent membrane insertion of GluA1 and GluA2 in the hippocampus as well as a rather surprising hippocampal synaptic potentiation [10, 11]. According to our current model, the ketamine induced synaptic potentiation results in a shift in excitation—inhibition balance towards more excitation. This premise is based on our finding that the synaptic potentiation strictly requires AMPA receptor function, involves an increase in AMPA receptor surface expression [11] and cannot be elicited by manipulation of inhibitory neurotransmission alone [10]. The resulting increase in activity elicits activation of CaMKII leading to MeCP2 Ser421 phosphorylation and subsequent alterations in gene expression that maintains and renders subsequent ketamine application more potent in triggering synaptic potentiation. Indeed, in this study, we also observed that potentiation of hippocampal responses following perfusion of ketamine was augmented (metaplasticity i.e., plasticity of plasticity) in hippocampal slices from animals that received a single low dose injection of ketamine 7 days prior compared to animals that received saline. We also observed that MeCP2 Ser421 phosphorylation was required for this repeated ketamine induced metaplasticity. Taken together, our findings suggest that acute ketamine facilitates the responsiveness to subsequent ketamine administration through this metaplasticity process.

## Future directions

Recent studies from our group as well as others suggest that specific pharmacological treatments for mood disorders activate acute and/or chronic homeostatic synaptic plasticity mechanisms to exert their behavioral effects [44, 54, 66–69]. While this review focused on the rapid homeostatic synaptic upscaling produced by ketamine, recent data has shown lithium in a slower-acting process can produce homeostatic synaptic downscaling, highlighting the diverse and complex effects psychoactive compounds can exert on homeostatic plasticity mechanisms [53]. Moreover, deficits in homeostatic synaptic plasticity have been shown in a variety of disease models of neuropsychiatric, neurological and neurodevelopmental disorders that include but not limited to Alzheimer’s Disease [70, 71], Rett Syndrome [72], and Fragile X [73]. In drug addiction, incubation of drug craving is proposed to involve a maladaptive form of homeostatic plasticity [74, 75]. It is important to note that homeostatic synaptic plasticities and their deficiencies have been reported in multiple systems, including the invertebrate neuromuscular junction and the mammalian visual cortex, demonstrating the evolutionary conserved nature of these processes and their potential role in the disease pathophysiology. Despite the growing evidence for the role of these forms of plasticities in disease pathology as well as treatment, homeostatic plasticity mechanisms as a direct drug target have received limited attention. In this context, studies on ketamine action reveal internally consistent and molecularly specific pathway that account for ketamine’s acute effects as well as long-term efficacy. Therefore, it is likely that the mechanisms exposed by ketamine action may serve as a Rosetta Stone for understanding the action of other potential treatments with rapid onset of therapeutic activity as exemplified by the recent enthusiasm surrounding psychedelic drugs [76], which may involve a form of AMPA receptor mediated plasticity reminiscent of ketamine action [77]. Overall, we convey strong optimism that tapping into homeostatic plasticity mechanisms in drug discovery will foster the development of novel treatments and synapse specific manipulations providing a path for versatile therapeutics with advantageous properties.

## References

[1] Malenka RC, Bear MF (2004). LTP and LTD: an embarrassment of riches. Neuron.

[2] Cannon WB, Rosenblueth A. The supersensitivity of denervated structures: a law of denervation (Macmillan, New York, 1949).

[3] Axelsson J, Thesleff S (1959). A study of supersensitivity in denervated mammalian skeletal muscle. J Physiol.

[4] Turrigiano GG, Leslie KR, Desai NS, Rutherford LC, Nelson SB (1998). Activity-dependent scaling of quantal amplitude in neocortical neurons. Nature.

[5] O’Brien RJ, Kamboj S, Ehlers MD, Rosen KR, Fischbach GD, Huganir RL (1998). Activity-dependent modulation of synaptic AMPA receptor accumulation. Neuron.

[6] Wierenga CJ, Ibata K, Turrigiano GG (2005). Postsynaptic expression of homeostatic plasticity at neocortical synapses. J Neurosci.

[7] Arendt KL, Zhang Y, Jurado S, Malenka RC, Südhof TC, Chen L (2015). Retinoic acid and LTP recruit postsynaptic AMPA receptors using distinct SNARE-dependent mechanisms. Neuron.

[8] Sutton MA, Ito HT, Cressy P, Kempf C, Woo JC, Schuman EM (2006). Miniature neurotransmission stabilizes synaptic function via tonic suppression of local dendritic protein synthesis. Cell.

[9] Turrigiano GG (2017). The dialectic of Hebb and homeostasis. Philos Trans R Soc Lond B Biol Sci..

[10] Autry AE, Adachi M, Nosyreva E, Na ES, Los MF, Cheng PF (2011). NMDA receptor blockade at rest triggers rapid behavioural antidepressant responses. Nature.

[11] Nosyreva E, Szabla K, Autry AE, Ryazanov AG, Monteggia LM, Kavalali ET (2013). Acute suppression of spontaneous neurotransmission drives synaptic potentiation. J Neurosci.

[12] Fatt P, Katz B (1950). Some observations on biological noise. Nature.

[13] Fatt P, Katz B (1952). Spontaneous subthreshold activity at motor nerve endings. J Physiol.

[14] Kavalali ET (2015). The mechanisms and functions of spontaneous neurotransmitter release. Nat Rev Neurosci.

[15] Wang C, Kavalali ET, Monteggia LM (2022). BDNF signaling in context: from synaptic regulation to psychiatric disease. Cell.

[16] Espinosa F, Kavalali ET (2009). NMDA receptor activation by spontaneous glutamatergic neurotransmission. J Neurophysiol.

[17] Reese AL, Kavalali ET. Spontaneous neurotransmission signals through store-driven Ca2+ transients to maintain synaptic homeostasis. *eLife*. 2015; 10.7554/eLife.09262. 10.7554/eLife.09262 PMC4534843 26208337

[18] Murthy VN, Schikorski T, Stevens CF, Zhu Y (2001). Inactivity produces increases in neurotransmitter release and synapse size. Neuron.

[19] Jakawich SK, Nasser HB, Strong MJ, McCartney AJ, Perez AS, Rakesh N (2010). Local presynaptic activity gates homeostatic changes in presynaptic function driven by dendritic BDNF synthesis. Neuron.

[20] Lindskog M, Li L, Groth RD, Poburko D, Thiagarajan TC, Han X (2010). Postsynaptic GluA1 enables acute retrograde enhancement of presynaptic function to coordinate adaptation to synaptic inactivity. Proc Natl Acad Sci USA.

[21] Tsodyks MV, Markram H (1997). The neural code between neocortical pyramidal neurons depends on neurotransmitter release probability. Proc Natl Acad Sci USA.

[22] Turrigiano GG, Nelson SB (2004). Homeostatic plasticity in the developing nervous system. Nat Rev Neurosci.

[23] Delvendahl I, Kita K, Müller M (2019). Rapid and sustained homeostatic control of presynaptic exocytosis at a central synapse. Proc Natl Acad Sci USA.

[24] Wondolowski J, Dickman D (2013). Emerging links between homeostatic synaptic plasticity and neurological disease. Front Cell Neurosci.

[25] Kim YI, Lømo T, Lupa MT, Thesleff S (1984). Miniature end-plate potentials in rat skeletal muscle poisoned with botulinum toxin. J Physiol.

[26] Washbourne P, Thompson PM, Carta M, Costa ET, Mathews JR, Lopez-Benditó G (2002). Genetic ablation of the t-SNARE SNAP-25 distinguishes mechanisms of neuroexocytosis. Nat Neurosci.

[27] Piedras-Rentería ES, Pyle JL, Diehn M, Glickfeld LL, Harata CN, Cao Y (2004). Presynaptic homeostasis at CNS nerve terminals compensates for lack of a key Ca2+ entry pathway. Proc Natl Acad Sci USA.

[28] Crawford DC, Ramirez DMO, Trauterman B, Monteggia LM, Kavalali ET (2017). Selective molecular impairment of spontaneous neurotransmission modulates synaptic efficacy. Nat Commun.

[29] Hengen KB, Torrado Pacheco A, McGregor JN, Van Hooser SD, Turrigiano GG (2016). Neuronal firing rate homeostasis is inhibited by sleep and promoted by wake. Cell.

[30] Ibata K, Sun Q, Turrigiano GG (2008). Rapid synaptic scaling induced by changes in postsynaptic firing. Neuron.

[31] Li B, Suutari BS, Sun SD, Luo Z, Wei C, Chenouard N (2020). Neuronal inactivity Co-opts LTP machinery to drive potassium channel splicing and homeostatic spike widening. Cell.

[32] Atasoy D, Ertunc M, Moulder KL, Blackwell J, Chung C, Su J (2008). Spontaneous and evoked glutamate release activates two populations of NMDA receptors with limited overlap. J Neurosci.

[33] Sara Y, Bal M, Adachi M, Monteggia LM, Kavalali ET (2011). Use-dependent AMPA receptor block reveals segregation of spontaneous and evoked glutamatergic neurotransmission. J Neurosci.

[34] Melom JE, Akbergenova Y, Gavornik JP, Littleton JT (2013). Spontaneous and evoked release are independently regulated at individual active zones. J Neurosci.

[35] Peled ES, Newman ZL, Isacoff EY (2014). Evoked and spontaneous transmission favored by distinct sets of synapses. Curr Biol.

[36] Reese AL, Kavalali ET. Single synapse evaluation of the postsynaptic NMDA receptors targeted by evoked and spontaneous neurotransmission. eLife 2016; 10.7554/eLife.21170. 10.7554/eLife.21170 PMC5148599 27882871

[37] Horvath PM, Piazza MK, Monteggia LM, Kavalali ET (2020). Spontaneous and evoked neurotransmission are partially segregated at inhibitory synapses. eLife.

[38] Wang CS, Chanaday NL, Monteggia LM, Kavalali ET (2022). Probing the segregation of evoked and spontaneous neurotransmission via photobleaching and recovery of a fluorescent glutamate sensor. eLife.

[39] Gonzalez-Islas C, Bülow P, Wenner P (2018). Regulation of synaptic scaling by action potential-independent miniature neurotransmission. J Neurosci Res.

[40] Ramirez DMO, Crawford DC, Chanaday NL, Trauterman B, Monteggia LM, Kavalali ET (2017). Loss of Doc2-dependent spontaneous neurotransmission augments glutamatergic synaptic strength. J Neurosci.

[41] Fong MF, Newman JP, Potter SM, Wenner P. Upward synaptic scaling is dependent on neurotransmission rather than spiking. Nat. Commun. 2015;6:6339. 10.1038/ncomms7339 PMC4355957 25751516

[42] Garcia-Bereguiain MA, Gonzalez-Islas C, Lindsly C, Wenner P (2016). Spontaneous release regulates synaptic scaling in the embryonic spinal network in vivo. J. Neurosci.

[43] Gideons ES, Lin PY, Mahgoub M, Kavalali ET, Monteggia LM (2017). Chronic lithium treatment elicits its antimanic effects via BDNF-TrkB dependent synaptic downscaling. eLife.

[44] Kavalali ET (2020). Neuronal Ca2+ signalling at rest and during spontaneous neurotransmission. J Physiol.

[45] Styr B, Gonen N, Zarhin D, Ruggiero A, Atsmon R, Gazit N (2019). Mitochondrial regulation of the hippocampal firing rate set point and seizure susceptibility. Neuron.

[46] Schaukowitch K, Reese AL, Kim SK, Kilaru G, Joo JY, Kavalali ET (2017). An intrinsic transcriptional program underlying synaptic scaling during activity suppression. Cell Rep.

[47] Kavalali ET, Plummer MR (1994). Selective potentiation of a novel calcium channel in rat hippocampal neurones. J Physiol.

[48] Kavalali ET, Plummer MR (1996). Multiple voltage-dependent mechanisms potentiate calcium channel activity in hippocampal neurons. J Neurosci.

[49] Kavalali ET, Zhuo M, Bito H, Tsien RW (1997). Dendritic Ca2+ channels characterized by recordings from isolated hippocampal dendritic segments. Neuron.

[50] Wang HL, Zhang Z, Hintze M, Chen L (2011). Decrease in calcium concentration triggers neuronal retinoic acid synthesis during homeostatic synaptic plasticity. J Neurosci.

[51] Suzuki K, Nosyreva E, Hunt KW, Kavalali ET, Monteggia LM (2017). Effects of a ketamine metabolite on synaptic NMDAR function. Nature.

[52] Arendt KL, Zhang Z, Ganesan S, Hintze M, Shin MM, Tang Y (2015). Calcineurin mediates homeostatic synaptic plasticity by regulating retinoic acid synthesis. Proc Natl Acad Sci USA.

[53] Kavalali ET, Monteggia LM (2020). Targeting homeostatic synaptic plasticity for treatment of mood disorders. Neuron.

[54] Sutton MA, Taylor AM, Ito HT, Pham A, Schuman EM (2007). Postsynaptic decoding of neural activity: eEF2 as a biochemical sensor coupling miniature synaptic transmission to local protein synthesis. Neuron.

[55] Aoto J, Nam CI, Poon MM, Ting P, Chen L (2008). Synaptic signaling by all-trans retinoic acid in homeostatic synaptic plasticity. Neuron.

[56] Sarti F, Schroeder J, Aoto J, Chen L (2012). Conditional RARalpha knockout mice reveal acute requirement for retinoic acid and RARalpha in homeostatic plasticity. Front Mol Neurosci.

[57] Poon MM, Chen L (2008). Retinoic acid-gated sequence-specific translational control by RARalpha. Proc Natl Acad Sci USA.

[58] Suzuki K, Kim JW, Nosyreva E, Kavalali ET, Monteggia LM (2021). Convergence of distinct signaling pathways on synaptic scaling to trigger rapid antidepressant action. Cell Rep.

[59] Maeng S, Zarate CA, Du J, Schloesser RJ, McCammon J, Chen G (2008). Cellular mechanisms underlying the antidepressant effects of ketamine: role of alpha-amino-3-hydroxy-5-methylisoxazole-4-propionic acid receptors. Biol Psychiatry.

[60] Cull-Candy S, Kelly L, Farrant M (2006). Regulation of Ca2+-permeable AMPA receptors: synaptic plasticity and beyond. Curr Opin Neurobiol.

[61] Wohleb ES, Gerhard D, Thomas A, Duman RS (2017). Molecular and cellular mechanisms of rapid-acting antidepressants ketamine and scopolamine. Curr Neuropharmacol.

[62] Widman AJ, McMahon LL (2018). Disinhibition of CA1 pyramidal cells by low-dose ketamine and other antagonists with rapid antidepressant efficacy. Proc Natl Acad Sci USA.

[63] Povysheva NV, Johnson JW (2016). Effects of memantine on the excitation-inhibition balance in prefrontal cortex. Neurobiol Dis.

[64] Gideons ES, Kavalali ET, Monteggia LM (2014). Mechanisms underlying differential effectiveness of memantine and ketamine in rapid antidepressant responses. Proc Natl Acad Sci USA.

[65] Kim JW, Autry AE, Na ES, Adachi M, Bjorkholm C, Kavalali ET (2021). Sustained effects of rapidly-acting antidepressants require BDNF-dependent MeCP2 phosphorylation. Nat Neurosci.

[66] Kim JW, Herz J, Kavalali ET, Monteggia LM (2021). A key requirement for synaptic Reelin signaling in ketamine-mediated behavioral and synaptic action. Proc Natl Acad Sci USA.

[67] Horvath PM, Chanaday NL, Alten B, Kavalali ET, Monteggia LM (2021). A subthreshold synaptic mechanism regulating BDNF expression and resting synaptic strength. Cell Rep.

[68] Lin PY, Ma ZZ, Mahgoup M, Kavalali ET, Monteggia LM (2021). A synaptic locus for TrkB signaling underlying ketamine rapid antidepressant action. Cell Rep.

[69] Suzuki K, Kavalali ET, Monteggia LM (2022). Optical analysis of AMPAR-mediated synaptic scaling in mouse hippocampus. STAR Protoc.

[70] Frere S, Slutsky I (2018). Alzheimer’s disease: from firing instability to homeostasis network collapse. Neuron.

[71] Styr B, Slutsky I (2018). Imbalance between firing homeostasis and synaptic plasticity drives early-phase Alzheimer’s disease. Nat Neurosci.

[72] Blackman MP, Djukic B, Nelson SB, Turrigiano GG (2012). A critical and cell-autonomous role for MeCP2 in synaptic scaling up. J Neurosci.

[73] Soden ME, Chen L (2012). Fragile X protein FMRP is required for homeostatic plasticity and regulation of synaptic strength by retinoic acid. J Neurosci.

[74] Conrad KL, Tseng KY, Uejima JL, Reimers JM, Heng LJ, Shaham Y (2008). Formation of accumbens GluR2-lacking AMPA receptors mediates. Nature.

[75] Wolf ME (2016). Synaptic mechanisms underlying persistent cocaine craving. Nat Rev Neurosci.

[76] McClure-Begley TD, Roth BL. The promises and perils of psychedelic pharmacology for psychiatry. Nat Rev Drug Discov. 2022; 10.1038/s41573-022-00421-7. 10.1038/s41573-022-00421-7 35301459

[77] Hesselgrave N, Troppoli TA, Wulff AB, Cole AB, Thompson SM (2021). Harnessing psilocybin: antidepressant-like behavioral and synaptic actions of psilocybin are independent of 5-HT2R activation in mice. Proc Natl Acad Sci USA.

